# Closed-loop systems for plants expressing animal proteins: a modernized framework to safeguard the future of agricultural innovation

**DOI:** 10.3389/fpls.2025.1426290

**Published:** 2025-01-30

**Authors:** Kristin A. Bresnahan, Justin M. Ferber, J. Thomas Carrato, Thomas J. Stoddard, Patrick V. Palad, Magi Richani

**Affiliations:** ^1^ Alpine Bio, South San Francisco, CA, United States; ^2^ Creative Biotech Solutions Limited Liability Corporation (LLC), Monterey, CA, United States

**Keywords:** closed-loop systems, plant molecular farming, biotechnology, stewardship, food allergen, alternative proteins

## Abstract

Escalating population growth and climate change pressures on crop production necessitate agricultural innovation to ensure food security and sustainability. Plant molecular farming (PMF), which uses genetically modified (GM) plants to produce high-value proteins for food products, offers a promising solution. PMF products, particularly those that express an animal protein in seed and grain crops, have the potential to substantially benefit U.S. and global agriculture, food systems, economies, and the environment. Farmers can diversify and generate increased revenue streams, while consumers gain access to affordable proteins beyond those currently available. However, the development and commercialization of PMF products, especially those expressing allergenic animal proteins, require careful consideration of existing stewardship guidelines and best practices. Current GM plant stewardship practices must be thoroughly assessed to identify and address any gaps, ensuring that PMF products maintain identity preservation and containment throughout their lifecycle. Implementing a fit-for-purpose closed-loop system (CLS) is crucial for effectively identifying, managing, and mitigating the potential risks and liabilities associated with PMF product development, production, and processing. A CLS framework for PMF products expressing animal proteins should integrate existing best practices from Excellence Through Stewardship and applicable third-party guidelines, including by way of example ISO standards, Good Manufacturing Practices, Hazard Analysis and Risk-Based Preventive Controls, Hazard Analysis and Critical Control Points, and Safe Quality Food, as well as newly designed controls to address potential PMF-specific risks. This comprehensive approach maximizes containment, identity preservation, regulatory compliance, traceability, incident response capabilities, and continuous improvement across the product lifecycle. While customization is required based on each PMF product developer’s specific product and operations, this paper examines industry best practices and describes CLS components that a PMF developer should consider in designing a robust, bespoke CLS to maintain identity preservation and product containment. Such a system will optimize for product quality and integrity while preventing commingling with commodity supplies and any associated market disruption while also addressing food safety. By implementing these rigorous systems, the PMF industry can realize its potential to contribute significantly to sustainable agriculture and food security.

## 1 Introduction

Innovation is crucial to America’s agricultural economy. As population growth and climate change pressure traditional food systems, genetically modified (GM) plants developed to produce high-value proteins, known as “plant-molecular farming” (PMF), offer potential for substantial positive impact on agricultural and food systems, economies, and the environment.

PMF products can efficiently and sustainably produce protein ingredients that traditionally come from resource-intensive systems. The potential benefits are wide-ranging, including to farmers through new value-added crops (Kunkler and Gerlt, 2024), businesses and consumers through scalable and affordable protein production (Vianna et al., 2011; Tschofen et al., 2016; Long et al., 2022; Dietz and Muldoon-Jacobs, 2024; Messina and Messina, 2024), and governments and society through increased food security with a lower climate impact (Mariotti, 2017; Lucas et al., 2023).

To achieve these benefits at scale, PMF developers should implement closed-loop systems (CLS) to manage potential risks and liabilities and safely commercialize their products, including preserving genetic identity, preventing commingling with commodity crops, avoiding allergen cross-contamination, protecting human health, and maintaining regulatory compliance. While current industry guidelines have successfully maintained GM crop identity and coexistence with conventional crops, they may need enhancement for certain PMF products (Tusé et al., 2024). The most effective approach leverages existing practices as a foundation, including, for example, Excellence Through Stewardship (ETS), ISO, Good Manufacturing Practices (GMPs) and food safety protocols. Where appropriate, these should be augmented with new practices, controls, and technologies to create a comprehensive stewardship program capable of managing potential PMF-specific risks. The CLS stewardship of PMF products discussed herein makes suggestions for PMF products that express animal proteins in seed and grain crops that are used for human food and animal feed. Any PMF products that fall outside of this category will also need to be stewarded in a CLS, but may have distinct and different considerations from what is discussed below.

## 2 Understanding potential risk to inform CLS development

Constructing an effective CLS first requires a PMF developer to thoroughly evaluate the potential risks (i.e. magnitude of hazard multiplied by the probability of the hazard) associated with each phase of its value chain, from research and development (“R&D”) through processing. This evaluation should begin with a comprehensive risk assessment that identifies critical hazards and impacts, as well as threat modeling. Once these potential risks have been identified, analyzed and ranked (e.g., scores based on inherent risk before mitigations are applied, as well as residual risk, or risk that remains after controls and mitigation strategies have been applied), a PMF developer will be better positioned to design and implement appropriate strategies and controls to manage or eliminate such potential risks. Moreover, regular performance of a risk assessment can enable a PMF developer to continue to reassess the potential risks across its business and the effectiveness of its CLS in managing or otherwise mitigating these potential risks. Regular evaluation of potential risk will also enable the developer to continuously improve and refine its CLS through the different phases of growth and scale of its business.

## 3 CLS principles for the responsible commercialization of PMF products expressing animal proteins

### 3.1 Current industry best practices and third-party guidelines

Building a comprehensive CLS for PMF products should start with existing agricultural and food safety best practices. These practices establish a proven basis for stewardship, containment, identity preservation, safety, regulatory compliance, and risk management. By leveraging existing industry best practices from programs such as Excellence Through Stewardship (ETS), International Organization for Standardization (ISO), Good Manufacturing Practices (GMPs), Hazard Analysis and Risk-Based Preventive Controls (HARPC), Hazard Analysis and Critical Control Points (HACCP), and Safe Quality Food (SQF), a PMF developer can accelerate the development of an effective CLS covering the full product lifecycle.

Current stewardship guidelines for GM crops, notably ETS and ISO, provide sophisticated requirements for managing stewardship from R&D through devitalization. However, for certain PMF products, such as PMF products developed to produce an allergenic animal protein, developers must build their CLS to encompass downstream processing activities in order to guard against potential allergen cross-contamination and commingling risks, and to ensure accountability for materials produced under the CLS (see, e.g., FDA, 2023).

### 3.2 Core CLS components and current industry best practices for R&D through processing

While specific CLS components will depend on each PMF developer’s products and operations, certain core elements described by ETS and ISO should form the foundation for a CLS for any PMF product, as summarized below. In addition, a PMF developer should also consider any and all applicable federal or state laws, rules or regulations.

*Risk Assessments*: Conduct regular risk assessments covering the product life cycle to quantify potential risks and inform mitigation strategies (ISO, 2015).

*Traceability/Inventory System*: Deploy an inventory management system designed to maintain product integrity, purity and traceability through the value chain, including mass balance of material handed off across the supply chain, labeling, tracking, and disposition of material (ETS, 2021).

*R&D Activities in the Lab*: Implement controls to prevent and address errors affecting product integrity during research and development (ETS, 2021).

*R&D Activities in Contained Facilities/Greenhouses*: Implement controls to prevent or manage errors involving product integrity; unintended cross pollination; inadvertent mixing, mislabeling, or improper disposition of seed or plant materials; and escape of seed or plant material from the facilities via human or other means (ETS, 2021).

*Field Plantings*: Implement appropriate controls, including considerations around plot design, planting location, appropriate isolation and buffer zones, and standard operating procedures (SOPs) to maintain product integrity and confinement in outdoor field plantings and ensure that the required activities are performed in accordance with those SOPs (ETS, 2021).

*Post-Harvest Management*: Conduct testing for genetic identity and purity, utilize clear labeling to identify harvested plant material, and utilize appropriate storage and packaging to prevent any loss of containment. Determine appropriate post-harvest land use restrictions for the land used to grow the PMF products (ETS, 2021).

*Testing*: Develop and validate detection methods to evaluate purity, identity preservation, and traceability throughout the value chain, including at critical control points (ETS, 2021).

*Product Devitalization/Destruction*: Establish and validate processes for devitalization/destruction of PMF materials prior to disposal (ETS, 2021).

*Restricted Access*: Implement controls to restrict access to sites and material to authorized personnel at each stage of the value chain where viable PMF plant materials are being developed, grown, stored, or processed.

*Vendor Assessment and Contracting*: Establish a comprehensive vendor assessment program and contracting process to vet third parties accessing PMF materials. Contracts with third parties should include specific terms requiring compliance with the PMF developer’s SOPs and CLS requirements (ETS, 2022).

*Training*: Develop and perform rigorous training programs for personnel and third parties handling PMF plant materials (ETS, 2021). Training should be specific to the activities at each phase of the value chain and delivered in advance of such activities. Ensure relevant personnel are trained when corrective measures or procedural changes are necessary (ETS, 2021).

*Record Keeping:* Create and securely retain comprehensive records of assessments, training and activities throughout the product development lifecycle (ETS, 2021).

*Ongoing Auditing and Monitoring*: Perform regular auditing and monitoring of operations, including third-party service providers and in-season and post-harvest volunteer monitoring and management (ETS, 2021). These activities should be driven by the output from risk assessments to ensure focus on the identified critical hazard and control points (ISO, 2015).

*Processes for Incident Response and Product Recall*: Design and implement an incident response plan and a product recall plan for potential incidents during each stage of the product life cycle (ETS, 2019). Key components of the incident response plan should include defined roles and accountabilities, process flows, established communication channels, documentation requirements, ongoing training, and a framework for corrective action planning.

*Hazard Analysis and Risk-Based Verifiable Controls for Food Safety Hazards*: Perform a hazard analysis to identify and evaluate the food safety hazards in grain, ingredient, or food processing facilities. Establish effective risk-based and verifiable controls at CCPs to ensure food safety (*see, e.g.*, Food and Drug Administration 21 CFR Part 117, Subpart C).

*SQF Food Safety Code*: Adhere to SQF codes to comply with accredited food safety standards. SQF develops food safety codes for food processors that is consistent with the Global Food Safety Initiative (GFSI) and provides stringent requirements for GMPs, product testing, processing, and allergen management (SQF, 2020).

Taken together, a combination of the above systems, processes and controls form the foundation of current industry best practice for CLS and food safety programs for PMF products expressing animal proteins intended for human consumption.

The next section will address the additional controls, processes, activities and technologies that can be layered on top of this foundation across the value chain – from field crop production activities through processing – to further assess and manage the potential risks specific to the development and commercialization of PMF products.

### 3.3 Building a CLS for PMF products expressing animal proteins with additional controls, processes, activities and technologies

The development of PMF products, particularly those producing known allergenic proteins, introduces challenges that are not necessarily contemplated by the current paradigm of industry best practices and third-party guidelines. Therefore, a tailored approach should be developed by layering processes, controls, and technologies on top of current industry best practices and guidelines to address the potential risks associated with the development and commercialization of those PMF products. This approach ensures a robust and comprehensive framework for PMF products, thereby enabling their safe, responsible, and sustainable production.

The CLS components supplementing the existing stewardship components described above should be applied across the value chain, from field crop production activities through processing,^1^ and in the case of products containing a known allergen, should take into account the safety of any workers that might be exposed to that allergen during the product life cycle depending on the nature of the products and how they are processed. The specific controls applied for any PMF product will depend on the developer’s operations, product characteristics, and the crop and trait. Each developer will need to consider the appropriate combination of additional processes, controls and technologies, in designing a comprehensive CLS to ensure identity preservation and containment of the PMF product.

The processes and controls described below are examples based on a PMF product developed in a food crop expressing an animal protein that is a known food allergen.

#### 3.3.1 On-farm crop production and field operations

*Crop Selection*: Consider the appropriate crop species for the development of PMF products, including the biology of the crop species, its sexual compatibility, seed dispersal, dormancy, and vegetative reproductive mechanisms, and how those factors impact potential risk. Factors like the species’ method of pollination (e.g. self-pollination versus wind or insect pollination) and the propensity for outcrossing can greatly impact biosafety and will inform the controls necessary to ensure containment.

*Isolation and Buffer Zones*: Implement appropriate isolation and buffer zones to avoid mechanical commingling (including appropriate equipment turnaround and cleanout areas) or cross-pollination based on crop, trait and risk assessment. Exact distances will depend on field selection, the crop’s reproductive biology and the target trait.

*Management of Crop Production*: Implement detailed SOPs to direct growers’ activities, from pre-planting through harvest, and in the following growing season, and require contractual obligations that cover all stewardship requirements, including delivery of harvested material and disposition of unused seed.

*Equipment and Storage*: Consider use of equipment dedicated for the duration of use with the PMF product based on the crop and trait risk assessment. Equipment that has direct contact with PMF materials that are viable should be dedicated for use with the PMF developer’s crop for the duration of those activities to prevent mixing of materials. Storage facilities for dedicated equipment and for plant material should be dedicated to the PMF developer's crop for their duration of use with those products, where feasible, and, regardless of whether dedicated storage is used, dedicated equipment should be properly secured and safeguarded. To the extent that dedicated equipment and storage facilities are not used, such equipment and facilities may require labeling until they are thoroughly cleaned and verified clean, e.g.: “This [equipment][facility] has been used in the handling or production of [PMF material].”

*Equipment Selection and Cleaning*: Given the rigorous cleaning required of equipment coming into direct contact with viable PMF material, equipment utilized for PMF material in the field should be assessed for feasibility of cleaning. For equipment that comes into direct contact with viable PMF material, the PMF developer should implement detailed equipment cleanout SOPs managed by trained personnel and specific to each piece of equipment. These SOPs should include clear, concise instructions to perform the cleaning and the post-cleanout verification processes, each performed by different personnel and documented with date and time for auditing purposes.

*Post-Harvest Land Use*: Consider appropriate requirements for subsequent rotation crops to facilitate monitoring for and termination of volunteers and to mitigate potential risks of contamination. One example might include the planting of a morphologically distinct non-sexually compatible crop with differing herbicide resistance from the PMF crop.

*Additional Monitoring and Performance Auditing Activities*: Consider appropriate additional onsite oversight and performance auditing of field operations, to be performed either by its full-time employees or trained third-party representatives. These additional monitoring activities should include in-season and post-harvest volunteer monitoring to avoid potential risk of commingling.

#### 3.3.2 Transportation and storage of viable PMF materials expressing animal proteins

Once the PMF materials have been harvested, they may be packaged and transported for further processing. For the transportation and storage of those PMF materials, developers should consider the implementation of additional components of the CLS discussed below.

*Transportation, Storage Facilities and Validated Cleaning Protocols*: The components of the transport activities (e.g., trailer, hopper or dedicated bins); equipment used to move bulk seed and grain (e.g. augers and conveyors); and storage facilities and containers (e.g. silos, bins, etc.) that come into direct contact with viable PMF material should be dedicated to the PMF developer for the duration of its use with that material. After that use is complete, the transport equipment and storage facilities and containers that came into direct contact with PMF material should be cleaned using a validated SOP and verified post-cleanout before being used for any other crops or materials.

*Additional Controls for Managing Post-Harvest Movement of PMF Material*: Consider additional controls to maintain visibility and control over movement, including new technologies. One example is real-time GPS tracking, which allows tracking of materials to ensure they are moving to intended locations and aids in incident management by enabling informed decision-making for containment and mitigation (see e.g., Nexyst 360, 2024). Another example is geo-fencing technology, which can manage and mitigate potential human error risks and potential commingling during transit by ensuring the storage containers cannot be opened outside of the specified geo-coordinates (see, e.g., Linxup, 2024).

*Detection Methods for Stored Material*: Deploy a rapid assay capable of confirming the identity of PMF material in storage.

#### 3.3.3 Processing of PMF materials expressing animal proteins

PMF materials developed to produce food ingredients, particularly those that include a known allergenic protein, present considerations that current industry best practices for the stewardship of GM crops do not fully contemplate, particularly during grain processing. In developing a CLS for PMF products expressing animal proteins that are a known allergen, a PMF developer should consider applicable food and worker safety standards and implement them to its processing activities to mitigate potential cross-contamination or commingling risks. The CLS components listed below should be considered for any PMF materials that include a known allergenic protein.

*Processing Contracts*: The contracts between the PMF developer and the processor should clearly spell out the requirements for identity preservation and segregation for PMF materials, processed fractions, co-products, and waste throughout the entire scope of processing activities. A PMF developer should work closely with its processors and monitor all critical activities to ensure they maintain the quality and integrity of products, co-products and waste.

*Storage*: While stored at the processing facility, viable and processed PMF materials should be stored in secure storage for the duration of its storage onsite. Before using the storage area for other materials, it should be thoroughly cleaned and verified to no longer contain viable and/or processed PMF material (see more under “Sanitation” below).

*Processing Lines and Equipment*: The processing lines and equipment used for PMF material should be dedicated during the period of use for such material. Processing lines and equipment should be thoroughly cleaned and tested for the presence of PMF materials before being used for other products or materials (see more under “Sanitation” below).

*Detection Methods*: Detection method(s) should enable the verifications of sanitation. While detection method sensitivities can vary, PMF developers should strive to find a method with the lowest achievable limit of detection (“LOD”) taking into account appropriate sampling size and method for the PMF product (e.g. protein). This method, including the LOD, should be validated on relevant equipment and material associated with processing activities.

*Sanitation*: PMF developers should work closely with processors to ensure that processing lines and equipment are cleaned according to the specified written requirements (i.e., sanitation program) before returning them for use with other products or materials. All options should be evaluated in designing a thorough sanitation program specific to the PMF material, including but not limited to cleaning and testing of applicable storage and processing equipment and verification requirements. One option for consideration is the purging of equipment with non-PMF material of the same species and testing the resulting output using a detection method with the lowest achievable LOD for the PMF product. Purge material should be managed within the CLS such that it does not enter the general supply chain.

*Waste Collection and Disposal*: Collect and manage waste from processing runs, including purge material if used, to ensure it is channeled properly in compliance with any regulatory requirements and any potential risks posed by the PMF product, and in a manner that does not present risk of cross contamination with other material in the typical value chain for that crop.

#### 3.3.4 Focus on continuous improvement and adapting with scale

Continuous improvement is critical for all quality management and stewardship systems, including any CLS developed for PMF products expressing animal proteins. As PMF products progress from R&D to precommercial to commercial launch and processing, CLS components must continue to evolve to remain effective. Every PMF developer should engage in continuous improvement and refinement of its CLS to maximize its effectiveness and manage potential risks posed at increasing operational scale and should require the same of its partners throughout its value chain.

It is also crucial that PMF developers share their learnings within the PMF community, fostering collective growth and improvement, as well as a shared set of industry principles and best practices for product stewardship. PMF developers should also share knowledge with and solicit feedback from relevant stakeholders across the agricultural value chain, including regulators, to ensure collaboration, awareness and transparency.

## 4 Conclusion

PMF products expressing animal proteins represent a significant innovation with the potential to positively impact agricultural and food systems, economies, and the environment. While current stewardship practices and guidelines provide a foundation, PMF developers should build comprehensive CLSs with components tailored to their operations and specific products to manage their potential risks. This approach will lead to containment, identity preservation, product integrity and purity, and food safety throughout the value chain. Continuous improvement of the CLS is also crucial for identifying and managing potential risks as a PMF developer’s business evolves. By developing and commercializing PMF products safely and responsibly, PMF developers will ensure the industry’s freedom to operate, and enable further innovation and growth.

Ultimately, it is the responsibility of every PMF developer to maintain product integrity, stewardship, and safety. Developers leading the way in PMF product development and commercialization provide a benefit to other developers and stakeholders by sharing their experiences and supporting the development of guidelines and best practices. This commitment will allow the industry to flourish while benefiting farmers, consumers, and the planet.

## Data Availability

The original contributions presented in the study are included in the article/supplementary material. Further inquiries can be directed to the corresponding author.

## Footnotes

1 While they should be reviewed in a risk assessment for the developer’s specific PMF product and operations, the processes and controls laid out by ETS and ISO are generally sufficient to cover activities in the lab and in contained plantings (i.e. for R&D activities taking place in enclosed spaces, like a laboratory or greenhouse) for PMF products. Therefore, those portions of the product life cycle will not be addressed in this section.
